# DPPH Radical Scavenging and Postprandial Hyperglycemia Inhibition Activities and Flavonoid Composition Analysis of Hawk Tea by UPLC-DAD and UPLC-Q/TOF MS^E^

**DOI:** 10.3390/molecules22101622

**Published:** 2017-10-13

**Authors:** Xuan Xiao, Lijia Xu, Huagang Hu, Yinjun Yang, Xinyao Zhang, Yong Peng, Peigen Xiao

**Affiliations:** 1 Key Laboratory of Bioactive Substances and Resources Utilization of Chinese Herbal Medicine, Ministry of Education, Institute of Medicinal Plant Development, Chinese Academy of Medical Sciences & Peking Union Medical College, Beijing 100193, China; xuanisle2008@163.com (X.X.); xulijia@hotmail.com (L.X.); iyangyinjun@163.com (Y.Y.); jvforever0@163.com (X.Z.); 2 Beijing Xiaotangshan Hospital, Xiaotangshan town, Beijing 102211, China; tangang_666@163.com

**Keywords:** Hawk tea, UPLC-DAD, UPLC-ESI-QTOF-MS, postprandial blood glucose, α-glucosidase, DPPH radicals scavenging and antimicrobial activities

## Abstract

Hawk tea (*Litsea coreana* Lévl. var. *Lanuginosa* (Migo) Yen C. Yang & P.H. Huang), a very popular herbal tea material, has attracted more and more attention due to its high antioxidant properties and possible therapeutic effect on type II diabetes mellitus. The raw materials of Hawk tea are usually divided into three kinds: bud tea (BT), primary leaf tea (PLT) and mature leaf tea (MLT). In this study, the DPPH radical scavenging activity and the antimicrobial properties of these three kinds of Hawk tea from different regions were comparatively investigated, and a ultra-high performance liquid chromatographic coupled with a photodiode array detector (UPLC-DAD) method was employed for comparison of the three major flavonoid constituents, including hyperoside, isoquercitrin and astragalin, in different samples of Hawk tea. At the same time, the effect of methanol extract (ME) of PLT on the mouse postprandial blood glucose and the effect of ME and its different fractions (petroleum ether fraction (PE), ethyl acetate fraction (EA), *n*-butanol fraction (*n*-BuOH), and water fraction (WF)) on the activity of α-glucosidase were studied. The results showed that Hawk BT and Hawk PLT possessed the higher radicals scavenging activity than Hawk MLT, while the antibacterial activity against *P. vulgaris* of PLT and MLT was higher than Hawk BT. The contents of the three major flavonoid constituents in samples of Hawk PLT are higher than Hawk BT and Hawk MLT. The mouse postprandial blood glucose levels of the middle dose (0.5 g/kg) group and the high dose (1 g/kg) group with oral administration of the ME of PLT were significantly lower than the control group. What’s more, the inhibitory effect of ME of PLT and its EA and *n*-BuOH fractions on α-glucosidase was significantly higher than that of acarbose. Rapid ultra-high performance liquid chromatography/quadrupole time-of-flight-mass spectrometry (UPLC-ESI-QTOF-MS) was used to identify the flavonoids in Hawk PLT, and a total of 20 flavonoids were identified or tentatively identified by comparing their retention times and accurate mass measurements with reference compounds or literature data. The bioactive flavonoid composition and DPPH radical scavenging activities present in different Hawk tea raw materials are quite different due to the different ontogenesis of these raw materials. Further studies on PLT showed that the substances in PLT ME could reduce the level of mouse postprandial blood glucose through inhibiting the activity of α-glucosidase.

## 1. Introduction

Natural antioxidant components in functional foods can protect the human body against the damage caused by free radicals, which may be the reason behind many chronic diseases [1]. The biological properties of these natural antioxidants are largely due to their high levels of various phenolic compounds, such as flavonoids and phenolic acids [2]. Currently, functional foods, such as herbal teas, have stepped into the public eye because of their beneficial properties for human health, and herbal teas have become a promising source of natural antioxidants [3,4,5].

Hawk tea is one of the most popular beverages with a long history in the southwest of China. It is produced from the buds or leaves of *Litsea coreana* Lévl. var. *Lanuginosa* (Migo) Yen C. Yang & P.H. Huang which is widely distributed in the mountainous area of the region [6], where Hawk tea is mainly produced in provinces such as Sichuan, Chongqing and Guizhou. Sichuan Province is the most important producing area and Hawk tea from Sichuan was sold all over the world.

In 2012, Hawk tea in Shimian County of Sichuan Province was awarded a protected product of national geography symbol by the General Administration of Quality Supervision, Inspection and Quarantine of the People’s Republic of China.

Hawk tea is not only a kind of drink, but also a traditional herbal medicine that could clear heat and relieve toxicity, cure swelling, and dissipate nodulation. It also can be used to invigorate the spleen, stimulate the appetite and warm the lungs to resolve phlegm according to the Compendium of Materia Medica [7]. Previous pharmacological studies have showed that Hawk tea had diverse benefits such as detoxication and detumescence, improvement of eyesight, antioxidation, reduction of blood lipids and fasting blood-glucose (FBG), and improvement of liver lipid metabolism [7,8,9]. According to recent reports, Hawk tea also had multiple biological activities, but the possible therapeutic effect on type II diabetes mellitus and antioxidant activity are of more interest to people [10,11,12].

Hawk tea usually is classified into three kinds: bud tea (BT), primary leaf tea (PLT), and mature leaf tea (MLT) according to the different harvest time and ontogenesis. Previous works just revealed the antioxidant activities and total flavonoid contents of the three kinds of Hawk tea which were harvested in the same place of Sichuan Province [9]. At the time, eight components in *Litsea coreana* Lévl. were identified as flavonoids (hyperin, isoquercitrin, quercitrin, quercetin, kaempferol), catechins, chlorogenic acid and epicatechin based on MS data and standard chromatographic characteristics [13]. However, the distribution of flavonoid compositions and individual flavonoid content in the three types of Hawk tea from different provinces and regions are still unknown. According to our investigation, Hawk tea infusions could be kept for three days and nights in summer during which the tea does not become rotten and sour, which suggests that Hawk tea may have microbial growth-inhibiting functions. Research also showed that volatile oils present in Hawk tea exhibited acceptable antimicrobial activities [12].

In this study, the antioxidant properties (DPPH radical scavenging activity) and the typical flavonoid contents of different types of Hawk tea from different geographical areas of the southwest of China were investigated and compared using in vitro assays and UPLC-DAD. The flavonoid compositions of Hawk tea were also systemically identified by UPLC-Q-TOF-MS^E^. The antimicrobial activity of Hawk tea was evaluated against some pathogenic bacteria. In addition, the effect of methanol extract (ME) of PLT on the mouse postprandial blood glucose and the effect of ME and its different extracts (ethyl acetate fraction (EA), *n*-butanol fraction (*n*-BuOH), and water fraction (WF)) on the activity of α-glucosidase were studied.

## 2. Results and Discussion

### 2.1. DPPH Radical Scavenging Activity

**Figure 1:**
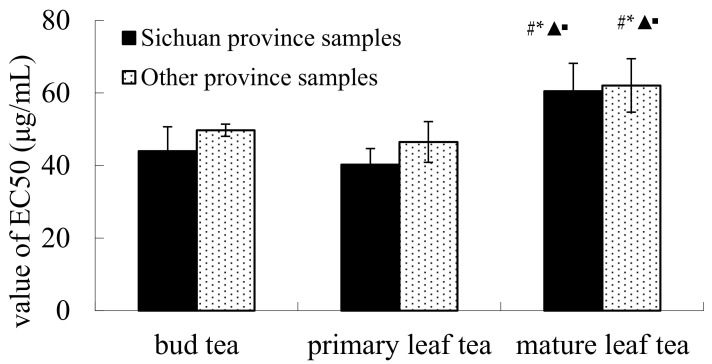
DPPH radicals scavenging activities of different kinds of Hawk tea. Note: vs. Sichuan bud tea group, # *p* < 0.05; vs. other province bud tea group, * *p* < 0.05; vs. Sichuan Hawk PLT group, ▲ *p* < 0.05; vs. other province Hawk PLT group, ▪ *p* < 0.05.

As shown in Figure 1, the EC_50_ values of all kinds of Hawk teas, including BT, PLT and MLT, from Sichuan Province were lower than that from other provinces. The EC_50_ values of MLT from Sichuan Province and other provinces were significantly higher than BT and PLT (*p* < 0.05). The results also showed that the DPPH radical scavenging activity of Hawk teas from Sichuan Province were higher than that from other provinces and that of BT and PLT were higher than that of MLT.

The production and scavenging of free radicals maintain a dynamic balance in human body [14]. However, excessive free radicals could induce damage to the cells or tissues and destroy the structure and function of biological macromolecules, which can in turn cause various diseases [15]. Natural antioxidants can scavenge free radicals to protect the health of the human body. The radical scavenging activity does not always correlate linearly with the capacity to inhibit oxidation of lipids or proteins [16], but to a certain extent, DPPH radical scavenging activity of an extract can reflect its potential antioxidant capacity in vivo. Hawk tea had been accepted by the public because of its high antioxidant activity and other health benefits [3].

A study conducted by Yuan et al. showed that Hawk primary leaf tea infusion (HPI) had the strongest DPPH radical scavenging activity, while Hawk mature leaf tea infusion (HMI) showed the lowest (*p* < 0.05) [10], so HMI was considered as the weakest electron donor among them. The general trend of our experimental results was similar to the results reported by Yuan, however, our study further found that the DPPH radical scavenging capacity of Hawk primary leaf tea (PLT) from Sichuan Province was the strongest in all kinds of Hawk tea sample.

### 2.2. Antibacterial Test

The in vitro antibacterial properties of BT, PLT and MLT are presented in Table 1. It is well known that the lower the minimum inhibitory concentration (MIC) value, the higher the antimicrobial activity. The tested extracts of BT, PLT and MLT possessed antibacterial activity against both Gram positive and Gram negative bacteria by the trace dilution method. The antimicrobial activity of Hawk tea against *S. aureus* and *B. subtilis* were already confirmed by Ji by the agar diffusion method [17]. This study illustrates the reasons from the perspective of inhibition of bacterial growth why Hawk tea infusions could be kept for three days and nights in summer while the tea does not become rotten and sour, and suggests that the Hawk tea could be used as a natural preservative against food spoilage.

The antibacterial activities of BT and PLT against *S. aureus* were significantly higher than MLT (*p* < 0.05) and that of PLT and MLT against *P. vulgaris* was significantly higher than BT (*p* < 0.05). The results showed that the antibacterial activity of Hawk teas might be different as a result of different bioactive components in Hawk teas.

### 2.3. The Effect on the Level of Postprandial Blood Glucose in Mice

The prevalence of diabetes mellitus in Chinese population was 11.6%, and has become one of major public health problems in China [18]. Research showed that oxidative stress is closely related to diabetes and also contributes to both the onset and the progression of chronic diabetic complications [19]. Oxidative stress could be defined as the increase of reactive oxygen species (ROS) generation. ROS include superoxide anion, hydrogen peroxide, hydroxyl radical and so on [20]. The ROS such as hydrogen peroxide arise from glucose metabolism [21]. Chronic exposure to high glucose concentrations could increase the mitochondrial metabolism through the hexosamine pathway, leading to overfull ROS production [20]. Persistent oxidative stress would impair pancreatic beta-cell function [21], and then lead to diabetic nephropathy [18], diabetic cardiomyopathy [22], diabetic retinopathy and other complications [23]. Recently, herbs having antioxidant activity have begun to be investigated as potential and adjunctive therapeutic agents for diabetes [18,24]. In our study, the DPPH radical scavenging activity of Sichuan Hawk PLT was the strongest in all kinds of Hawk tea sample, so the Hawk PLT sample S11, a kind of national geography symbol protected products from Shimian County of Sichuan Province which is the main producing area of Hawk tea, was chosen to be used in this test.

**Figure 2:**
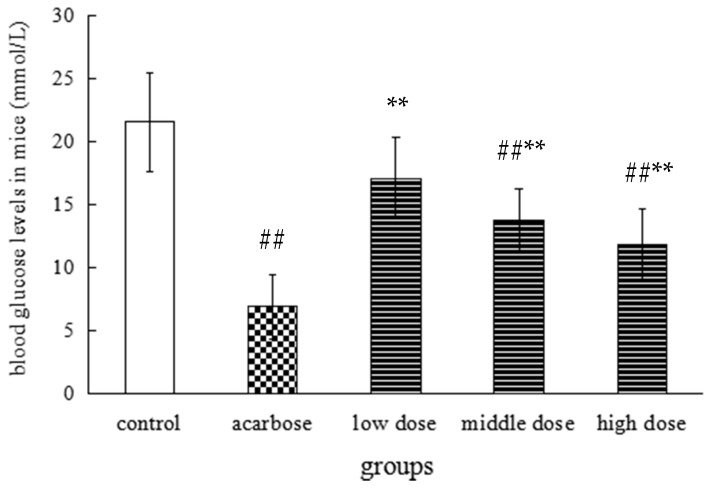
Effect of methanol extract from PLT on postprandial blood glucose levels of mice. Note: *n* = 8, vs. control group, ## *p* < 0.01; vs. acarbose group, ** *p* < 0.01.

Diabetes and pre-diabetes resulting in high blood glucose levels and hyperglycemia are divided into two cases: fasting high blood glucose and postprandial hyperglycemia [25]. Recent studies indicate that modulation of postprandial blood glucose plays an important role in long term glycemic control [26]. Measurement of postprandial blood glucose has also been widely practiced for monitoring of blood glucose [27]. Scientific research has showed that many foods or natural products could help regulate blood glucose levels. For instance, catechin in green tea, polyphenol in leaves of persimmon, and apple leaf extract have all been reported to inhibit the increase of blood glucose levels. In our study, the mouse postprandial blood glucose levels of the middle dose (0.5 g/kg) group and the high dose (1 g/kg) group with oral administration of the ME of PLT were significantly lower than the control group, but higher than the acarbose group (Figure 2). In addition, the total flavonoids in Hawk tea can significantly decrease the fasting blood glucose of type II diabetic rats [8]. These research results suggested that the extract of Hawk tea could decrease not only postprandial blood glucose, but also the fasting blood glucose.

### 2.4. α-Glucosidase Inhibitory Effect In Vitro

α-Glucosidase, which is an important enzyme for carbohydrate digestion, has been recognized as a therapeutic target for postprandial hyperglycemia and has received a lot of attention [28]. Glucosidase inhibitors can significantly reduce postprandial blood glucose levels in patients with type II diabetes mellitus and reduce diabetic complications [29]. A study conducted by Gou et al. showed that the ethanol extract of Hawk PLT could effectively inhibit the activity of α-glucosidase [30]. The impressive result obtained from our further study is that ME of PLT and its EA and *n*-BuOH fractions are inhibitors of α-glucosidase, and exhibited higher suppression than acarbose on α-glucosidase in vitro (as shown in Table 2). Another study also showed that puerh tea polysaccharide was an inhibitor of α-glycosidase three times more potent than acarbose which is now commonly used in the clinic as a hypoglycemic drug [31]. In this experiment, ME of PLT and its EA and *n*-BuOH fractions are mixtures, and these mixtures, which are composed of different monomers, may inhibit the activity of α-glucosidase in different ways, while the acarbose is just a monomer, so the in vitro α-glucosidase inhibitory activity of these three mixtures may be higher than that of acarbose. However, the postprandial blood glucose level in high dose group of mice fed ME of PLT is still higher than that of acarbose group according to the Figure 2. It may be due to the fact that some of compounds in the ME of PLT are metabolized into other components in mice. Therefore, the effect of ME of PLT on α-glucosidase in mice may be lower than that of acarbose.

These combined findings revealed that Hawk PLT could reduce the level of mouse postprandial blood glucose through inhibiting the activity of α-glucosidase. At the same time, Hawk tea may be a potential raw material for the development of α-glucosidase inhibitors.

### 2.5. Quantitative Analysis

#### 2.5.1. Optimization of the Extraction Method

For optimal extraction of the target compounds, different extraction solvents (30%, 75%, 100% methanol and 30% aqueous ethanol, 70% aqueous ethanol, 95% ethanol) were investigated in parallel, as well as different extraction times (30, 45 and 60 min). The peak areas of three target components (hyperoside, isoquercitrin and astragalin) were compared for more effective extraction. As a result, the optimum extraction method was the use of 75% methanol ultrasonic extraction for 60 min.

#### 2.5.2. Validation of Method

The linearity, limits of detection (LOD), limits of quantification (LOQ), accuracy and precision of the quantification of three analytes were validated. As shown in Table 3, the calibration curves of the analytes showed good linearity with high correlation coefficients (*R*^2^ > 0.9997) within the tested range. The limits of detection (LOD) and limits of quantification (LOQ) of the three analytes varied from 0.086 to 0.308 μg/mL and 0.025 to 0.922 μg/mL, respectively. The RSD of the precision of intra-and inter-day analysis ranged from 1.16% to 4.18% and 1.63% to 4.26%, respectively (Table 3). The mean recovery rates of all analytes were in the range of 99.9% to 105.6% with RSD less than 4.6%, which showed good reproducibility of the developed method (Table 3).

#### 2.5.3. Quantitative Analysis of Samples

**Figure 3:**
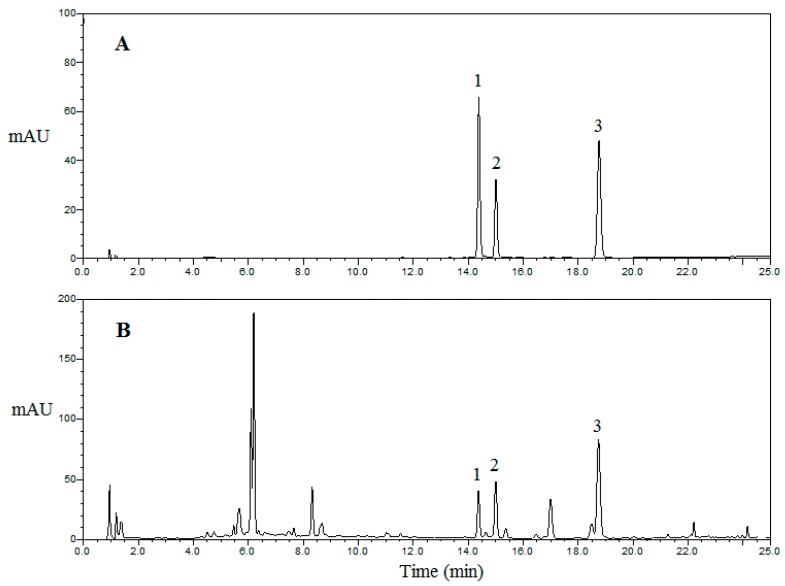
Typical UPLC chromatograms of mixed standards (**A**) and crude extract of Hawk primary leaf tea (PLT) (**B**): (1) hyperoside; (2) isoquercitrin; (3) astragalin. The detection wavelength was set at 280 nm.

The developed UPLC-DAD method was successfully applied for the simultaneous determination of hyperoside, isoquercitrin, and astragalin in Hawk teas of different kinds and from different regions. The chromatograms for standards and samples of different kinds of Hawk tea are shown in Figure 3. The contents of these compounds and their total contents are summarized in Table 4.

Due to the different kinds and regions of Hawk tea, there were remarkable differences between the samples in terms of the total content of investigated compounds, which ranged from 1.33 to 29.66 mg/g, as shown in Table 4. There were also significant differences among the three kinds of Hawk tea, in which total contents of the three monitored compounds in the PLT (10.47~29.66 mg/g) were much higher than BT (1.76~10.58 mg/g) and MLT (1.33~10.36 mg/g). On the other hand, the total content of the three compounds in Hawk tea showed no significant difference in four different regions, and among these the samples from Sichuan demonstrated higher levels than others. Among the three target compounds, the content of isoquercitrin (5.81 mg/g, 15.55 mg/g, and 5.61 mg/g in BT, PLT and MLT, respectively) was the highest in each sample.

Flavonoids, the main secondary metabolites in plants, are the most important quality-related compounds in herbs [32]. Flavonoids are the main group of polyphenols and they are important antioxidants due to their high redox potential [33]. The main group of flavonoids in Hawk tea are hyperoside, isoquercitrin, quercitrin, quercetin, kaempferol, catechins, chlorogenic acid, epicatechin, quercetin-3-*O*-β-d-galactopyranoside, kaempferol-3-*O*-β-d-glucopyranoside, kaempferol-3-*O*-β-d-galactopyranoside and quercetin-3-*O*-β-d-glucopyranoside [13,34]. Yu and Gu found that the content of total flavones in Hawk tea was three times higher than that in green tea [35]. Previous research showed that Hawk bud tea infusion (HBI) was the richest source of total flavonoids, followed by Hawk primary leaf tea infusion (HPI), while Hawk mature leaf tea infusion (HMI) was the lowest [10]. The Hawk tea materials in their experiment were collected from the same town in Sichuan Province, and BT, PLT and MLT were harvested in around February, March, and May, respectively.

The synthesis and accumulation of flavonoids occurs in response to environmental cues [36]. As a predominant secondary metabolic pathway in tea plants, flavonoid biosynthesis increases with increasing temperature and illumination by influencing structural gene expression or the activity of some enzymes [32,37]. In our study, the BT, PLT, and MLT were collected around March, May and July, respectively, and they were obtained from different provinces and regions. Our results could reflect the above rules. The research showed that the total amount of the three kinds of flavonoids in PLT was the highest, followed by BT, while the MLT showed the lowest levels.

The results of this study were different from those of the research conducted by Yuan et al. There may be two reasons for this phenomenon. Firstly, only the total amount of three kinds of flavonoids in our research was tested, while the total content of flavonoids was measured in the experiment conducted by Yuan et al. The experimental method used was different too. On the other hand, studies have shown that the flavonoid metabolic pathway was involved in the regulation mechanisms of plants to various stressful conditions [38]. Flavonoids are the main regulators of plant growth and defense, which are induced biosynthesis as the result of a long-term natural selection and acclimatization process [39,40,41,42]. The content of total flavonoids may be affected by region, climate, and maturity degree. The collection time and place of Hawk tea samples were completely different in the two researches. Therefore, the results of the two experiments were also different. However, a common trend was also found that the content of flavonoids in mature leaf tea (MLT) was the lowest among the three kinds of Hawk tea.

### 2.6. Identification of Flavonoids

**Figure 4:**
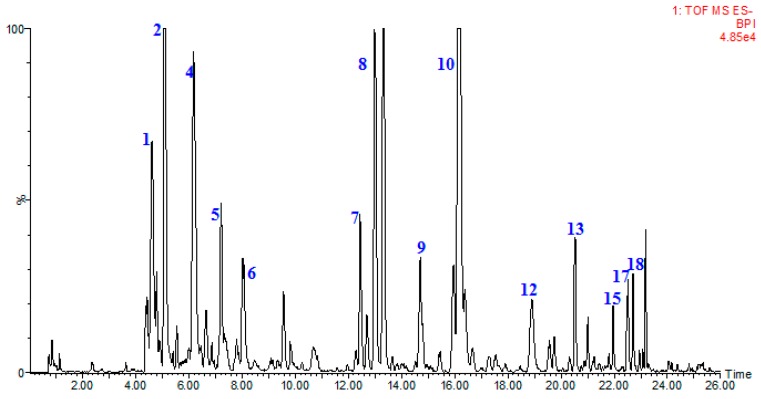
The base peak chromatograms of Hawk primary leaf tea by UPLC-ESI-QTOF-MS in negative ion mode.

Suitable chromatographic conditions were achieved through the optimization of mobile phase, elution program and conditions of mass. Under the present conditions, typical chromatogram of samples with mass spectrometric detection at negative ion mode was present in Figure 4. A total of 20 compounds were identified from the Hawk tea. Among them, five compounds were unambiguously identified by comparing their retention times, accurate masses and fragment ions with those of reference compounds and 15 compounds were tentatively assigned by matching the empirical molecular formula with that of known compounds previously reported in the literature and are presented in Table 5.

**Figure 5:**
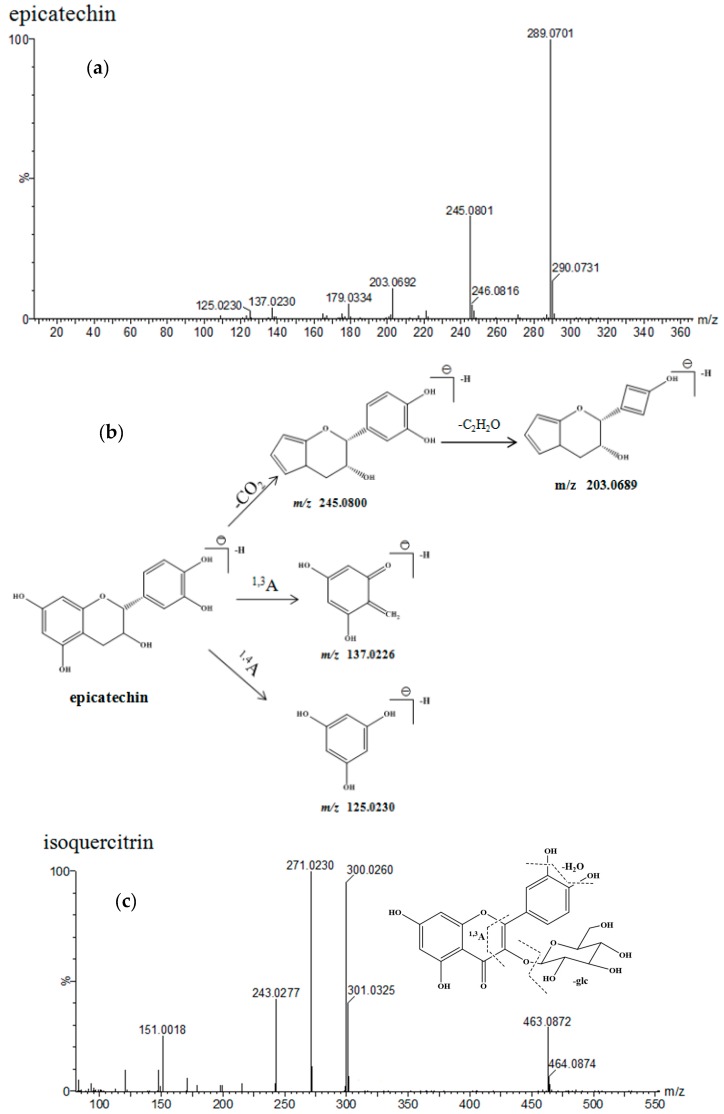

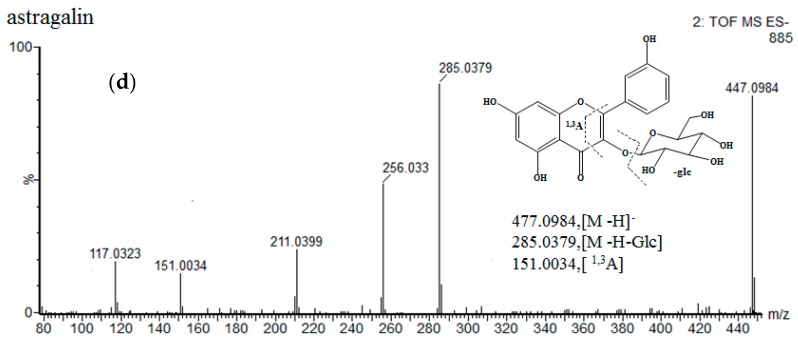
MS spectra and the proposed fragmentation pathway of epicatechin, isoquercitrin and astragalin, (**a**) epicatechin MS spectra; (**b**) the cleavage of epicatechin; (**c**) isoquercitrin MS spectra; (**d**) astragalin MS spectra.

Catechin (peak 2, 5.09 min) and epicatechin (peak 5, 7.21 min) all showed two major ultraviolet absorption bands at 190–220 nm and 278 nm. A major molecular ion at *m*/*z* 289.0701 [M − H]^−^ (calculated for C_15_H_14_O_6_, 289.0712) was observed in the mass spectrum. At high CE, four characteristic fragment ions at *m*/*z* 245.0801, 203.0692, 137.0230 and 125.0266 were observed due to the cleavage of covalent bond and retro-Diels-Alder rearrangement in ring C, which is shown in Figure 5 [43]. Likewise, peaks 1, 3, 4 and 6 all gave the same ultraviolet absorption bands at 190–220 nm and 278 nm, which showed that these compounds were all flavanols and their oligomers [44]. Among them, peaks 1, 3, 4 all showed [M − H]^−^ ions at *m/z* 577.1352, 577.1354 and 561.1401, corresponding to C_30_H_26_O_12_ and C_30_H_26_O_11_. According to their empirical molecular formula and literature report [45,46], peaks 1, 3 and 4 belonged to procyanidin dimers and were tentatively identified as epicatechin-(4-8)-epicatechin, epicatechin-(4-6)-epicatechin, epiafzelechin-(4-8)-epicatechin, respectively. Peak 6 showed a predominant molecular ion at *m/z* 883.2103 [M − H]^−^ in negative ion mode. Other fragment ions were found at *m*/*z* 561.1407, 543.1275, 407.0939, 289.0724, and 271.0651 indicating that the compound was possibly epiafzelechin-epiafzelechin-epicatechin.

Isoquercitrin (peak 8, 13.02 min) produced a minor protonated ion [M − H]^−^ at *m*/*z* 463.0877 and a dominant fragment ion [M − H − glucose]^−^ at *m/z* 301.0323 in negative mode. In addition, the fragment ion at *m*/*z* 151.0013 [M − H − glucose − H_2_O − C_8_H_8_O_2_]^−^ was produced by a retro-Diels-Alder rearrangement [47]. Astragalin (peak 10, 16.21 min) gave an [M − H]^−^ ion at *m*/*z* 447.0936, which was 16 Da less than isoquercitrin. Peaks 7, 9, 10, 11, 12, 13, 14, 15, and 16 all showed the same ultraviolet absorption bands as isoquercitrin and astragalin in 220–280 nm, 300–400 nm. Peaks 7, 9, 11, 15 and 16 produced a major ion at *m*/*z* 301.0323, indicating the flavonoid aglycone was quercetin. According to the other fragment ions, peaks 7, 9, 11, 15, and 16 were tentatively identified as hyperoside, quercitrin, quercetin-3-*O*-α-l-rhamnoside, quercetin-3-*O*-β-d-rutinose, and quercetin, respectively [48]. Peaks 12, 13 and 14 all gave ions at *m*/*z* 287.0551, and 285.0404, indicating their flavonoid aglycone was kaempferol. On the basis of the available reference data, peaks 12, 13 and 14 were tentatively identified as dihydrokaempferol-3-*O*-β-d-glucopyranoside, kaempferol-3-*O*-α-l-rhamnoside and dihydrokaempferol, respectively [48].

In addition, another type of compounds existed in Hawk tea. Peaks 17, 18, 19, 20 displayed a major ion [M − H]^−^ at *m*/*z* 593.1310, 595.1467, and in the MS^2^ fragmentation they lost a neutral fragment of 308 Da, yielding the fragment ion at *m*/*z* 285.0400, 287.0541. This ion showed typical fragments of kaempferol. The part of 308 Da was composed of a coumaroyl and glucopyranose according the fragment ions at high CE. Therefore, peaks 17, 18, 19, and 20 were tentatively identified as kaempferol-3-*O*-β-d-(6-*O*-*trans*-*p*-coumaroyl)-glucopyranoside, kaempferol-3-*O*-β-d-(6-*O*-*trans*-*p*-coumaroyl)-mannoside, dihydrokaempferol-3-*O*-β-d-(6-*O*-trans-*p*-coumaroyl)-glucopyranoside and dihydrokaempferol-3-*O*-β-d-(6-*O*-*trans*-*p*-coumaroyl)-mannoside, respectively [48,49].

## 3. Materials and Methods

### 3.1. Chemical and Reagents

Authentic standards of catechin, epicatechin, hyperoside, isoquercitrin and astragalin with purities above 95% as determined by LC analysis were purchased from the National Institutes for Food and Drug Control (Beijing, China). Acetonitrile (HPLC grade) and chromatographic grade methanol (MeOH) was obtained from Honeywell Burdick & Jackson (Muskegon, MI, USA). Chromatographic grade formic acid was purchased from CNW Technologies GmbH (Dusseldorf, Germany). Soluble corn starch, petroleum ether, ethyl acetate and *n*-butanol were obtained from Beijing Chemical Works (Beijing, China). The chemicals used in this study were of analytical or chromatographic grade. Baker’s yeast α-glucosidase (EC 3.2.1.20), *p*-nitrophenyl-α-d-gluco-pyranoside (pNPG), acarbose and 2,2-diphenyl-1-picrylhydrazyl (DPPH) were purchased from Sigma Chemical Co. (St. Louis, MO, USA). Ultra-pure water (*R* = 18 M Ω cm) used throughout the study was produced by a Milli-Q system (Millipore Corp, Billerica, MA, USA).

### 3.2. Plant Materials

The samples of Hawk teas (*Litsea coreana* Lévl. var. *Lanuginosa* (Migo) Yen C. Yang & P.H. Huang) were collected from Chongqing, Sichuan, Guizhou and Anhui province. The buds, primary leaves, and mature leaves of Hawk tea were harvested during the months of March, May and July 2014, respectively. The plant materials were authenticated by Peigen Xiao, and a voucher specimen (#20130715) was deposited in Institute of Medicinal Plant Development, Chinese Academy of Medical Science and Peking Union Medical College. The detailed information of the sample is listed in Table 6.

### 3.3. Bacterial Species

*Staphylococcus aureus* (ATCC 25923-3), *Bacillus cereus* (ATCC 11778-3), *Bacillus subtilis* (CMCC 63507-3), *Escherichia coli* (ATCC 25922-3), *Pseudomonas aeruginosa* (ATCC 27853-3) and *Proteus vulgaris* (CMCC 49027) were purchased from the China General Microbiological Culture Collection Center (Beijing, China).

### 3.4. Samples Preparation

All of material samples were dried in the shade and powdered. An accurately weighed amount of sample (0.5 g) was extracted twice with 75% methanol (50 mL) under ultrasound (300 w, 50 Hz) for 60 min. After dilution, the extract was filtered through a 0.22 µm filter and aliquots (2 µL) were injected into the chromatographic system for further analysis. The extract in DPPH scavenging assay and antibacterial assay was the same as that in UPLC-DAD and UPLC-Q/TOF-MS^E^ assay, while the extract in antibacterial assay was dried and dissolved in dimethylsulfoxide (DMSO).

The Hawk tea sample S11 was chosen to be used in the test of the postprandial blood glucose elevation in normal mice and α-glucosidase inhibitory effect in vitro. This sample S11 is a kind of national geography symbol protected product collected from Shanquan Village (Meiluo Town, Shimian County, Sichuan Province) which is the main Hawk tea-producing area. The methanol extract extraction method of sample S11 was the same as in the previous experiment. According to the method, Hawk PLT (500 g) was extracted, and all the methanol extracts were combined and filtered through filter paper, concentrated with a vacuum evaporator, and dried with a freeze drier to a powder. A part of the freeze-dried extract was dispersed with 20 vol (*v*/*w*) distilled water into a suspension and then fractionated with 1 vol (*v*/*v*) of petroleum ether, ethyl acetate and *n*-butanol, respectively. The fractionation with each solvent was repeated three times. Then the fractionated samples and the aqueous layer were concentrated and freeze-dried to afford the corresponding freeze-dried petroleum ether fraction (PE), ethyl acetate fraction (EA), *n*-butanol fraction (*n*-BuOH) and water fraction (WF).

### 3.5. Bioactivity Assay

#### 3.5.1. DPPH Radical Scavenging Assay

The DPPH radical scavenging assay was conducted according to the method described by Yasuda with minor modifications [50]. Briefly, DPPH solution (2 mL, 2 × 10^−4^ mol·L^−1^ in ethanol) was mixed with the samples dissolved in the extracting solvent (2 mL) at six different concentrations (0.0039–0.5 mg/mL). The reaction mixture was shaken, incubated in the dark at room temperature, and after 30 min the absorbance was read at 517 nm against a blank. Controls were prepared in a similar way as for the test group except for the replacement of the antioxidant solution with the corresponding extraction solvent. Results were expressed as concentration for 50% of maximal effect (EC_50_): Percent of disappearance = [(A_control_ − A_sample_)/A_control_] × 100%, where, A_control_ is the absorbance of control sample and A_sample_ is the absorbance of sample with the crude extract. And then the serial extract concentrations and the corresponding percents of DPPH disappearance were used to calculate the value of EC_50_ by the software GraphPad Prism v6.02 (GraphPad Software, La Jolla, CA, USA).

#### 3.5.2. Antibacterial Assay

The minimum inhibitory concentration (MIC) values were determined using the medium microdilution methodology according to Hector et al. with some modifications [4]. The assays were performed on 96 well plates with 100 μL of Mueller Hinton Broth (MHB), 100 μL of extract solutions and 5 μL of test bacterial suspensions at 1.0 × 10^7^ CFU mL^−1^. The extracts were dried and then dissolved in DMSO at concentration of 31.28 mg/mL to 0.24 mg/mL, and the incubation was made at 37 °C for 24 h. The bioactivities were recorded as blue coloration in the wells after the use of resazurin dye. The bacteriostatic or bactericidal effects of the extracts assayed were observed by inoculation of the well materials on Mueller Hinton Agar plates after the tests. For positive controls the antibiotic norflaxacin (5 mg/mL) and tetracycline (5 mg/mL) were used for all the tested strains. As for negative control the DMSO was used during the tests. Each assay was repeated three times.

#### 3.5.3. Blood Glucose Levels in Mice

This experiment on mice obtained the approval from an independent ethics committee at the Institute of Medicinal Plant Development, and the mice received humane care in compliance with international guidelines. Male Kunming mice (20 ± 2 g; SCXK 2012-0004; Experimental Animal Center of Academy of Military Medical Sciences, Beijing, China) were housed in the temperature controlled room (23 ± 2 °C) for 1 week before the experiment, and they could get water and food freely. After a 20 h-fast, blood glucose readings were determined from the tail vein of mice using One Touch Ultra Glucose Test Strips (Life Scan, Inc., Milpitas, CA, USA). Mice were divided into five groups (*n* = eight mice per group) at random, and there was no significant differences in initial blood glucose level of every group.

The soluble corn starch was dissolved in water (200 mg/mL), and then the ME of Hawk PLT was suspended in corn starch solution. The test groups were fed starch solution supplemented with 0.25 g/kg, 0.5 g/kg, and 1 g/kg (body weight, b.w.) of the ME of Hawk PLT respectively, acarbose was suspended in corn starch solution as positive control drug (0.1 g/kg, b.w.), while the control group mice were fed only the same the volume of corn starch solution through oral administration [51]. The blood samples were collected from the tail vein at 60 min after the last administration. And then blood glucose levels was measured using One Touch Ultra Glucose Test Strips.

#### 3.5.4. α-Glucosidase Inhibition Assasy

The α-glucosidase inhibitory activity of the Hawk tea was determined according to the method described by Apostolidis and Lee with a slight modification [51]. A mixture of samples of different concentrations (50 µL, acarbose, ME, PE, EA, *n*-BuOH, WF; 0.25 mg/mL, 0.5 mg/mL, 1 mg/mL) and 100 µL of 0.1 M phosphate buffer (pH 6.9) containing α-glucosidase solution (1 U/mL) was incubated in 96 well plates at 25 °C for 10 min. After preincubation, 50 µL of 5 mM pNPG solution in 0.1 M phosphate buffer (pH 6.9) was added into each well at timed intervals. The reaction mixtures were incubated at 25 °C for 5 min. Each sample was provided with 6 test repeat (*n* = six repeat per sample). Before and after incubation, absorbance was recorded at 405 nm by microplate reader (Benchepark Plus, Bio-Rad Laboratories, Inc., Hercules, CA, USA). Acarbose was used as the positive control. The α-glycosidase inhibitory activity was expressed as inhibition percent and was calculated as follows: Inhibitione (%) = [(A_control_ − A_sample_)/A_control_] × 100, where A_samp_ and A_control_ were defined as absorbance of the sample and the control, respectively.

#### 3.5.5. Statistical Analysis

Data were expressed as Mean ± standard deviation (SD). Statistical analysis was performed by SPSS 20.0 (SPSS Inc., Chicago, IL, USA). One-way analysis of variance (ANOVA) was utilized to evaluate differences, and *p* < 0.05 was regarded as statistically significant.

### 3.6. Equipment and Chromatographic Conditions

#### 3.6.1. UPLC-DAD Analysis Conditions

UPLC-DAD analyses were performed on an UltiMate 3000 system (DIONEX, Sunnyvale, CA USA) equipped with a pump, an autosampler, a column compartment and a diode array detector (DAD). Chromatographic separation was carried out on an ACQUITY UPLC HSS T3 column (2.1 × 100 mm, 1.8 µm, Waters Corp., Milford, MA, USA) at a column temperature of 30 °C, with a flow rate of 0.3 mL/min. Gradient elution of 0.1% formic acid solution (solvent A) and acetonitrile (solvent B) was employed: 0~2 min, 5% B; 2~12 min, 5~15% B; 12~20 min, 15~20% B; 20~25 min, 20~40% B; 25~30 min; 40~95% B. The injection volume was 5 µL, and the detection wave-length was set at 280 nm.

#### 3.6.2. UPLC-ESI-QTOF-MS Analysis Conditions

UPLC-ESI-QTOF-MS analysis was carried out on an Acquity UPLC™ system (Waters Corp.). The mobile phase was same to UPLC-DAD analysis, as well as the flow of rate and the injection volume. The tandem mass experiment was performed in negative ESI ionization modes with the data acquisition ranging of *m/z* 150–1000. The capillary voltage was optimized to 3.0 kV, and the cone voltage was 30 V. The source temperature was 120 °C, and the desolvation temperature was 350 °C. The desolvation gas flow was set to 600 L/h, and the cone gas flow was set at 50 L/h. Finally, the instrument was controlled by the Waters Masslynx 4.1 software.

### 3.7. Method Validation

#### 3.7.1. Linearity, Limits of Detection and Limits of Quantification

The calibration curves were constructed by at least six different concentrations of chemical markers, plotted by the peak area (Y) of analytes against concentration (X). Each concentration was analyzed in triplicate. The limits of detection (LOD) and limits of quantification (LOQ) were measured on the basis of the signal-to-noise ratio(S/N) of 3 and 10, respectively.

#### 3.7.2. Accuracy, Precision and Repeatability

The standard solution mixture was analyzed under the optimal conditions six times in one day for intra-day variation and on three successive days for inter-day variation. Then the relative standard deviation (RSD) was taken to evaluate the precision.

In order to check the repeatability, five different solutions made from the same sample were determined. The recovery test was used to evaluate the accuracy of this method by the formula: recovery (%) = (found amount − original amount)/spiked amount × 100%.

## 4. Conclusions

In summary, the antioxidant and the antimicrobial activities and the bioactive components of the three types of Hawk tea were comparatively investigated by DPPH assay, MIC values assay, and UPLC-DAD, respectively.

Research showed that BT and PLT possessed significantly higher DPPH radical scavenging activities and antibacterial activities against *S. aureus* than MLT. Also, PLT possessed higher content of major flavonoids. The results suggested that the DPPH radical scavenging and antimicrobial activities of Hawk tea may be significantly affected by its bioactive flavonoid components as a result of the ontogenesis of the raw material.

In our study, the mouse postprandial blood glucose levels with oral administration of the ME of PLT were significantly lower than the control group. Further study showed that ME of PLT and its EA and *n*-BuOH fractions are inhibitors of α-glucosidase, and exhibited higher suppression on α-glucosidase in vitro than acarbose. These studies suggest that the substances in ME of PLT could reduce the level of mouse postprandial blood through inhibition of the activity of α-glucosidase.

In addition, the chemical profile of one kind of Hawk tea, PLT, was systematically investigated by UPLC-QTOF/MS. Twenty flavonoids were identified or tentatively identified by comparing with references or literature data, providing a deeper understanding on the chemical constituents of Hawk tea.

## Figures and Tables

**Table 1:** Antimicrobial activity of Hawk tea extracts (MIC value expressed in mg/mL).

Sample	*S. aureus*	*B. subtilis*	*B. cereus*	*E. coli*	*P. aeruginosa*	*P. vulgaris*
Bud tea	3.48 ± 1.89	3.69 ± 1.81	4.56 ± 1.96	4.99 ± 2.78	4.78 ± 2.42	10.86 ± 5.79
Primary leaf tea	3.48 ± 0.86	3.26 ± 0.98	6.95 ± 1.72	3.91 ± 1.69	6.73 ± 2.20	6.52 ± 1.96 #
Mature leaf tea	5.65 ± 2.06 # *	5.43 ± 2.35	5.87 ± 2.39	6.08 ± 3.96	5.21 ± 2.59	4.78 ± 1.72 ##

Note: vs. bud tea sample, # *p* < 0.05, ## *p* < 0.01; vs. primary leaf tea sample, * *p* < 0.05.

**Table 2:** Inhibitory effect of Hawk primary leaf tea extracts on α-glucosidase.

Groups	α-Glucosidase Inhibitory Effect (%)		
	Low Concentrations (0.25 mg/mL)	Middle Concentrations (0.5 mg/mL)	High Concentrations (1 mg/mL)
Acarbose	51.71 ± 2.17	65.43 ± 3.50	73.94 ± 4.15
ME	72.19 ± 4.21 **	81.56 ± 3.11 **	88.23 ± 2.99 **
PE	19.56 ± 3.47 **^, ##^	41.24 ± 2.77 **^, ##^	54.87 ± 3.01 **^, ##^
EA	81.16 ± 4.33 **	86.37 ± 3.89 **	92.17 ± 3.67 **
*n*-BuOH	80.92 ± 2.08 **	84.16 ± 3.68 **	91.29 ± 3.43 **
WF	56.23 ± 4.26 ^##^	68.94 ± 3.54 ^##^	77.86 ± 2.91 ^##^

Note: *n* = 6, vs. acarbose group, ** *p* < 0.01; vs. ME group, ^##^ *p* < 0.01.

**Table 3:** Calibration curve, LOD and LOQ, and recovery for three standards.

Analytes	Linear Regression Equation of 3 Flavonoids			LOD (μg/mL)	LOQ (μg/mL)	Recovery (%) Mean ± SD
	Regressive Equation	*r*^2^	Test Range (μg/mL)			
Hyperoside	Y = 35.125X − 1.3227	0.9991	1.72–880.00	0.218	0.656	98.2 ± 2.35
Isoquercitrin	Y = 48.820X − 1.0970	0.9993	1.24–639.00	0.308	0.922	101.3 ± 1.78
Astragalin	Y = 12.930X − 0.6021	0.9984	0.36–360.00	0.086	0.257	103.1 ± 2.66

**Table 4:** The content of three flavonoids and their total content in Hawk tea (mean ± STDEV, *n* = 3).

Sample	Kinds	Hyperoside (mg/g)	Isoquercitrin (mg/g)	Astragalin (mg/g)	Total Content (mg/g)
S1	bud tea	1.62 ± 0.02	3.24 ± 0.11	1.18 ± 0.02	6.04
S2	bud tea	2.54 ± 0.04	5.81 ± 0.08	2.23 ± 0.04	10.58
S3	bud tea	0.81 ± 0.01	1.51 ± 0.02	0.56 ± 0.02	2.88
S4	bud tea	0.68 ± 0.02	1.49 ± 0.02	0.61 ± 0.003	2.78
S5	bud tea	0.95 ± 0.03	2.24 ± 0.04	0.45 ± 0.02	3.64
S6	bud tea	1.21 ± 0.01	4.39 ± 0.04	2.87 ± 0.02	8.47
S7	bud tea	0.75 ± 0.03	1.96 ± 0.01	0.78 ± 0.01	3.49
S8	bud tea	0.35 ± 0.002	1.62 ± 0.01	0.41 ± 0.02	2.38
S9	bud tea	0.41 ± 0.01	0.92 ± 0.02	0.43 ± 0.01	1.76
S10	primary leaf tea	2.19 ± 0.06	6.73 ± 0.17	5.01 ± 0.18	13.93
S11	primary leaf tea	5.54 ± 0.11	15.55 ± 0.24	8.57 ± 0.09	29.66
S12	primary leaf tea	2.34 ± 0.04	5.56 ± 0.16	2.57 ± 0.08	10.47
S13	primary leaf tea	3.89 ± 0.03	8.87 ± 0.01	3.01 ± 0.12	15.77
S14	primary leaf tea	4.15 ± 0.11	8.13 ± 0.07	3.12 ± 0.09	15.40
S15	primary leaf tea	3.05 ± 0.02	9.19 ± 0.11	6.82 ± 0.12	19.06
S16	primary leaf tea	1.69 ± 0.04	7.51 ± 0.07	3.95 ± 0.03	13.15
S17	primary leaf tea	2.09 ± 0.03	6.55 ± 0.11	4.83 ± 0.08	13.47
S18	primary leaf tea	6.48 ± 0.08	11.39 ± 0.14	7.80 ± 0.11	25.67
S19	mature leaf tea	1.28 ± 0.09	5.61 ± 0.12	3.47 ± 0.14	10.36
S20	mature leaf tea	0.75 ± 0.006	3.98 ± 0.03	2.21 ± 0.04	6.94
S21	mature leaf tea	0.66 ± 0.005	3.12 ± 0.02	2.82 ± 0.04	6.60
S22	mature leaf tea	0.67 ± 0.01	1.19 ± 0.03	0.74 ± 0.02	2.60
S23	mature leaf tea	0.67 ± 0.02	1.16 ± 0.01	0.31 ± 0.01	2.14
S24	mature leaf tea	0.72 ± 0.009	1.46 ± 0.02	0.86 ± 0.01	3.04
S25	mature leaf tea	0.71 ± 0.01	1.52 ± 0.03	0.67 ± 0.01	2.90
S26	mature leaf tea	0.37 ± 0.01	1.09 ± 0.06	0.32 ± 0.009	1.78
S27	mature leaf tea	0.32 ± 0.009	0.67 ± 0.02	0.34 ± 0.01	1.33

**Table 5:** Identification of 20 compounds detected in Hawk primary leaf tea by UPLC-Q-TOF-MS.

No.	Identification	Rt. (min)	UV λmax (nm)	Formula	Quasi-Molecular ES-			(ES-)MS^E^ Ions (*m/z*)
					Calc. Mass	Measured Mass	Error (ppm)	
1	epicatechin-(4-8)-epicatechin	4.63	203 279	C_30_H_26_O_12_	577.1346	577.1352 [M − H]^−^	1	425.0876, 407.0754, 289.0692, 245.0478
2	catechin	5.09	203 278	C_15_H_14_O_6_	289.0712	289.0711 [M − H]^−^	−0.7	245.0816, 203.0692, 137.0236
3	epicatechin-(4-6)-epicatechin	5.82	201 279	C_30_H_26_O_12_	577.1346	577.1354 [M − H]^−^	1.4	425.0876, 407.0779, 289.0707, 245.0478
4	epiafzelechin-(4-8)-epicatechin	6.19	198 278	C_30_H_26_O_11_	561.1397	561.1401 [M − H]^−^−	0.7	425.0872, 407.0774, 289.0714, 271.0599
5	epicatechin	7.21	202 278	C_15_H_14_O_6_	289.0712	289.0717 [M − H]^−^	1.7	245.0802, 203.0689, 137.0156
6	epiafzelechin-epiafzelechin-epicatechin	8.03	196 278	C_45_H_38_O_16_	833.2082	833.2103 [M − H]^−^	2.5	561.1407, 543.1275, 407.0939, 289.0724, 271.0651
7	hyperoside	12.45	254 354	C_21_H_20_O_12_	463.0877	463.0877 [M − H]^−^	−0.4	301.0302, 151.0022
8	isoquercitrin	13.02	255 353	C_21_H_20_O_12_	463.0877	463.0878 [M − H]^−^	0.2	301.0323, 151.0018
9	quercitrin	14.70	264 347	C_21_H_20_O_11_	447.0927	447.0929 [M − H]^−^	0.4	301.0318, 151.0016
10	astragalin	16.21	264 346	C_21_H_20_O_11_	447.0927	447.0936 [M − H]^−^	2	285.0398, 151.0018
11	quercetin-3-*O*-α-l-rhamnoside	16.37	255 348	C_21_H_20_O_11_	447.0927	447.0911 [M − H]^−^	−3.6	301.0337, 151.0027
12	dihydrokaempferol-3-*O*-β-d-glucopyranoside	18.62	199	C_21_H_22_O_11_	449.1084	449.1073 [M − H]^−^	−2.4	287.0551, 151.0028
13	kaempferol-3-*O*-α-l-rhamnoside	20.51	212 264	C_21_H_20_O_10_	431.0978	431.0983 [M − H]^−^	1.2	285.0404, 151.0017
14	dihydrokaempferol	21.72	199 287	C_15_H_12_O_6_	287.0556.	287.0543 [M − H]^−^	−4.5	151.0017, 135.0436
15	quercetin-3-*O*-β-d-rutinose	21.95	255 248	C_30_H_26_O_14_	609.1244	609.1246 [M − H]^−^	0.2	463.0860, 301.0325, 151.0017
16	quercetin	22.24	255 347	C_15_H_10_O_7_	301.0348	301.0330 [M − H]^−^	−1.8	285.0398, 151.0018
17	kaempferol-3-*O*-β-d-(6-*O*-*trans*-*p*-coumaroyl)-glucopyranoside	22.51	266 313	C_30_H_26_O_13_	593.1295	593.1310 [M − H]^−^	2.5	447.0961, 285.0400, 151.0016
18	kaempferol-3-*O*-β-d-(6-*O*-*trans*-*p*-coumaroyl)-mannoside	22.69	266 312	C_30_H_26_O_13_	593.1295	593.1310 [M − H]^−^	2.5	447.0961, 285.0400, 151.0016
19	dihydrokaempferol-3-*O*-β-d-(6-*O*-*trans*-*p*-coumaroyl)-glucopyranoside	23.49	266 313	C_30_H_28_O_13_	595.1452	595.1467 [M − H]^−^	0.8	449.1109, 287.0541, 151.0020
20	dihydrokaempferol-3-*O*-β-d-(6-*O*-*trans*-*p*-coumaroyl)-mannoside	24.31	266 313	C_30_H_28_O_13_	595.1452	595.1467 [M − H]^−^	0.8	449.1109, 287.0541, 151.0020

**Table 6:** Region, kind and collection time information of the samples.

Samples	Region	Kinds	Harvesting Time
S1	Pingtou Village, Meiluo Town, Shimian County, Sichuan province	Bud tea	March 2014
S2	Shanquan Village, Meiluo Town, Shimian County, Sichuan province	Bud tea	March 2014
S3	Liuhe Village, Cheling Town, Mingshan County, Sichuan province	Bud tea	March 2014
S4	Chapingli Village, Chaba Town, Qingchuan County, Sichuan province	Bud tea	March 2014
S5	Xinshi Village, Wawushan Town, Hongya County, Sichuan province	Bud tea	March 2014
S6	Anlezhai Village, Anle Town, Jiuzhaigou County, Sichuan province	Bud tea	March 2014
S7	Chunxiao Village, Nanling Town, Wushan County, Chongqing	Bud tea	March 2014
S8	Longfeng Village, Xinglong Town, Meitan County, Guizhou province	Bud tea	March 2014
S9	Hong Village, Fangtang Town, Ningguo County, Anhui province	Bud tea	March 2014
S10	Pingtou Village, Meiluo Town, Shimian County, Sichuan province	Primary leaf tea	May 2014
S11	Shanquan Village, Meiluo Town, Shimian County, Sichuan province	Primary leaf tea	May 2014
S12	Liuhe Village, Cheling Town, Mingshan County, Sichuan province	Primary leaf tea	May 2014
S13	Chapingli Village, Chaba Town, Qingchuan County, Sichuan province	Primary leaf tea	May 2014
S14	Xinshi Village, Wawushan Town, Hongya County, Sichuan province	Primary leaf tea	May 2014
S15	Anlezhai Village, Anle Town, Jiuzhaigou County, Sichuan province	Primary leaf tea	May 2014
S16	Chunxiao Village, Nanling Town, Wushan County, Chongqing	Primary leaf tea	May 2014
S17	Longfeng Village, Xinglong Town, Meitan County, Guizhou province	Primary leaf tea	May 2014
S18	Hong Village, Fangtang Town, Ningguo County, Anhui province	Primary leaf tea	May 2014
S19	Pingtou Village, Meiluo Town, Shimian County, Sichuan province	Mature leaf tea	July 2014
S20	Shanquan Village, Meiluo Town, Shimian County, Sichuan province	Mature leaf tea	July 2014
S21	Liuhe Village, Cheling Town, Mingshan County, Sichuan province	Mature leaf tea	July 2014
S22	Chapingli Village, Chaba Town, Qingchuan County, Sichuan province	Mature leaf tea	July 2014
S23	Xinshi Village, Wawushan Town, Hongya County, Sichuan province	Mature leaf tea	July 2014
S24	Anlezhai Village, Anle Town, Jiuzhaigou County, Sichuan province	Mature leaf tea	July 2014
S25	Chunxiao Village, Nanling Town, Wushan County, Chongqing	Mature leaf tea	July 2014
S26	Longfeng Village, Xinglong Town, Meitan County, Guizhou province	Mature leaf tea	July 2014
S27	Hong Village, Fangtang Town, Ningguo County, Anhui province	Mature leaf tea	July 2014

## References

[1] Zheng W., Wang S.Y. (2001). Antioxidant activity and phenolic compounds in selected herbs. J. Agric. Food Chem..

[2] Garzón G.A., Narváez C.E., Riedl K.M., Schwartz S.J. (2010). Chemical composition, anthocyanins, non-anthocyanin phenolics and antioxidant activity of wild bilberry (*Vaccinium meridionale* Swartz) from Colombia. Food Chem..

[3] Aoshima H., Hirata S., Ayabe S. (2007). Antioxidative and anti-hydrogen peroxide activities of various herbal teas. Food Chem..

[4] Hector H.F.K., Felipe M.A.d.S., Fábio C.G., Antonia Q.L.D.S., Afonso D.L.D.S. (2013). Antioxidant, antimicrobial activities and characterization of phenolic compounds from buriti (*Mauritia flexuosa* L.f.) by UPLC-ESI-MS/MS. Food Res. Int..

[5] Chen L.X., Hu D.J., Lam S.C., Ge L., Wu D., Zhao J., Long Z.R., Yang W.J., Fan B., Li S.P. (2016). Comparison of antioxidant activities of different parts from snow chrysanthemum (*Coreopsis tinctoria* Nutt.) and identification of their natural antioxidants using high performance liquid chromatography coupled with diode array detection and mass spectrometry and 2,2′-azinobis(3-ethylbenzthiazoline-sulfonic acid)diammonium salt-based assay. J. Chromatogr. A.

[6] Jia X.J., Ding C.B., Yuan S., Zhang Z.W., Chen Y.E., Du L., Yuan M. (2014). Extraction, purification and characterization of polysaccharides from Hawk tea. Carbohydr. Polym..

[7] Wang J.Q., Li J., Zou Y.H., Cheng W.M., Lu C., Zhang L., Ge J.F., Huang C., Jin Y., Lv X.W. (2009). Preventive effects of total flavonoids of *Litsea coreana* leve on hepatic steatosis in rats fed with high fat diet. J. Ethnopharmacol..

[8] Sun Y.X., Lu Y.X., Wang L.Y., Li D.D., Zhang Q., Zhang L.Z., Yang F., Li J. (2010). Study on the mechanism of action of total flavonoids of *Litsea coreana* for reducing blood glucose level in rat with type 2 diabetes mellitus. Chin. J. Integr. Tradit. West. Med..

[9] Ye M., Liu D., Zhang R., Yang L., Wang J. (2012). Effect of hawk tea (*Litsea coreana* L.) on the numbers of lactic acid bacteria and flavour compounds of yoghurt. Int. Dairy J..

[10] Yuan M., Jia X.J., Ding C.B., Yuan S., Zhang Z.W., Chen Y.E. (2014). Comparative studies on bioactive constituents in Hawk tea infusions with different maturity degree and their antioxidant activities. Sci. World J..

[11] Jia X.J., Dong L.H., Yang Y., Yuan S., Zhang Z.W., Yuan M. (2013). Preliminary structural characterization and antioxidant activities of polysaccharides extracted from Hawk tea (*Litsea coreana* var. *Lanuginosa*). Carbohydr. Polym..

[12] Yu B., Zhang D., Yan X.W., Wang J.W., Yao L., Tan L.H., Zhao S.P., Li N., Cao W.G. (2016). Comparative Evaluation of the Chemical Composition, Antioxidant and Antimicrobial Activities of the Volatile Oils of Hawk Tea from Six Botanical Origins. Chem. Biodivers..

[13] Meng Q., Qian Z.M., Li X.X., Li D.Q., Huang W.H., Zhao J., Li S.Q. (2012). Free radical scavenging activity of Eagle tea and their flavonoids. Acta Pharm. Sin. B.

[14] Young I.S., Woodside J.V. (2001). Antioxidants in health and disease. J. Clin. Pathol..

[15] Deetae P., Parichanon P., Trakunleewatthana P., Chanseetis C., Lertsiri S. (2012). Antioxidant and anti-glycation properties of Thai herbal teas in comparison with conventional teas. Food Chem..

[16] Morita M., Naito Y.J., Yoshikawa T., Niki E. (2017). Antioxidant capacity of blueberry extracts: Peroxyl radical scavenging and inhibition of plasma lipid oxidation induced by multiple oxidants. J. Berry Res..

[17] Ji H.F., Zhang L.W., Zhang L., Wang Q., Zhang H.R. (2011). Microwave-assisted extraction and antibacterial bioactivity of total flavonoids from *Litsea coreana* L.. J. Henan Inst. Sci. Technol..

[18] Han H.Y., Cao A., Wang L., Guo H.J., Zang Y.J., Li Z.Z., Zhang X.M., Peng W. (2017). Huangqi Decoction Ameliorates Streptozotocin-Induced Rat Diabetic Nephropathy through Antioxidant and Regulation of the TGF-β/MAPK/PPAR-γ Signaling. Cell. Physiol. Biochem..

[19] Rains J.L., Jain S.K. (2011). Oxidative stress, insulin signaling, and diabetes. Free Radic. Biol. Med..

[20] Chaudhuri A., Umpierrez G.E. (2012). Oxidative stress and inflammation in hyperglycemic crises and resolution with insulin: Implications for the acute and chronic complications of hyperglycemia. J. Diabetes Complicat..

[21] Pi J., Zhang Q., Fu J., Woods C.G., Hou Y., Corkey B.E., Collins S., Andersen M.E. (2010). ROS signaling, oxidative stress and Nrf2 in pancreatic beta-cell function. Toxicol. Appl. Pharmacol..

[22] Guido M.C., Marques A.F., Tavares E.R., Tavares de Melo M.D., Salemi V.M.C., Maranhão R.C. (2017). The Effects of Diabetes Induction on the Rat Heart: Differences in Oxidative Stress, Inflammatory Cells, and Fibrosis between Subendocardial and Interstitial Myocardial Areas. Oxidative Med. Cell. Longev..

[23] Zhang X., Zhao J., Zhao T., Liu H. (2015). Effects of intensive glycemic control in ocular complications in patients with type 2 diabetes: A meta-analysis of randomized clinical trials. Endocrine.

[24] Tsai F.J., Li T.M., Ko C.H., Cheng C.F., Ho T.J., Liu X., Tsang H.Y., Lin T.H., Liao C.C., Li J.P. (2017). Effects of Chinese herbal medicines on the occurrence of diabetic retinopathy in type 2 diabetes patients and protection of ARPE-19 retina cells by inhibiting oxidative stress. Oncotarget.

[25] Forbes J.M., Cooper M.E. (2013). Mechanisms of diabetic complications. Physiol. Rev..

[26] Herath H.M.M., Weerarathna T.P., Fonseka C.L., Vidanagamage A.S. (2017). Targeting postprandial blood sugar over fasting blood sugar: A clinic based comparative study. Diabetes Metab. Syndr..

[27] Inzucchi S.E., Bergenstal R.M., Buse J.B., Diamant M., Ferrannini E., Nauck M., Peters A.L., Tsapas A., Wender R., Matthews D.R. (2015). Management of hyperglycemia in type 2 diabetes, 2015: A patient-centered approach: Update to a position statement of the American Diabetes Association and the European Association for the Study of Diabetes. Diabetes Care.

[28] Krentz A.J., Bailey C.J. (2005). Oral antidiabetic agents: Current role in type 2 diabetes mellitus. Drugs.

[29] Zhen J., Dai Y., Villani T., Giurleo D., Simon J.E., Wu Q. (2017). Synthesis of novel flavonoid alkaloids as α-glucosidase inhibitors. Bioorg. Med. Chem..

[30] Gou L., Zhong Y.Y., Yang Y., Wang X.Y. (2016). Inhibitory effect of ethanol extract from Hawk tea on α-glucosidase. J. Xiamen Univ. (Nat. Sci.).

[31] Xu P., Wu J., Zhang Y., Chen H., Wang Y.F. (2014). Physicochemical characterization of puerh tea polysaccharides and their antioxidant and α-glycosidase inhibition. J. Funct. Foods.

[32] Zhang Q.F., Liu M.Y., Ruan J.Y. (2017). Metabolomics analysis reveals the metabolic and functional roles of flavonoids in light-sensitive tea leaves. BMC Plant Biol..

[33] Tsao R., Yang R. (2003). Optimization of a new mobile phase to know the complex and real polyphenolic composition: Towards a total phenolic index using high-performance liquid chromatography. J. Chromatogr. A.

[34] Chen Y.P., Cheng W.M., Li J. (2008). Analyse on the chemical constituents from flavonoids of *Litsea coreana* L.. Acta Univ. Med. Anhui.

[35] Yu J.P., Gu L.Q. (2001). The chemical constituent of laoying Tea from Guizhou. J. Plant Resour. Environ..

[36] Cheynier V., Comte G., Davies K.M., Lattanzio V., Martens S. (2013). Plant phenolics: Recent advances on their biosynthesis, genetics, and ecophysiology. Plant Physiol. Biochem..

[37] Figueiredo-González M., Cancho-Grande B., Boso S., Santiago J.L., Martínez M.C., Simal-Gándara J. (2013). Evolution of flavonoids in Mouratón berries taken from both bunch halves. Food Chem..

[38] Agati G., Biricolti S., Guidi L., Ferrini F., Fini A., Tattini M. (2011). The biosynthesis of flavonoids is enhanced similarly by UV radiation and root zone salinity in *L. vulgare* leaves. J. Plant Physiol..

[39] Tattini M., Galardi C., Pinelli P., Massai R., Remorini D., Agati G. (2004). Differential accumulation of flavonoids and hydroxycinnamates in leaves of *Ligustrum vulgare* under excess light and drought stress. New Phytol..

[40] Agati G., Azzarello E., Pollastri S., Tattini M. (2012). Flavonoids as antioxidants in plants: Location and functional significance. Plant Sci..

[41] Gould K., Tattini M., Jay-Allemand C. (2015). New evidences for the functional roles of secondary metabolites in plant-environment interactions. Environ. Exp. Bot..

[42] Landi M., Tattini M., Gould K.S. (2015). Multiple functional roles of anthocyanins in plant-environment interactions. Environ. Exp. Bot..

[43] Liu G.Q., Dong J., Wang H., Wan L.R., Duan Y.S., Chen S.Z. (2009). ESI fragmentation studies of four tea catechins. Chem. J. Chin. Univ..

[44] Hümmer W., Schreier P. (2008). Analysis of proanthocyanidins. Mol. Nutr. Food Res..

[45] Chen Y., Wang B., Yu J.N., Li B.L., Wen Y., Liu R., Tan J. (2013). Separation and identification of A-type and B-type dimers of procyanidins from Peanut Skin by RP-HPLC-ESI-MS/MS. Food Sci..

[46] Fraser K., Collette V., Hancock K.R. (2016). Characterization of proanthocyanidins from seeds of perennial ryegrass (*Lolium perenne* L.) and tall fescue (*Festuca arundinacea*) by liquid chromatography-Mass spectrometry. J. Agric. Food Chem..

[47] Lin Z., Yang R., Tang Y.N. (2014). LC-MS/MS analysis of eight kinds of flavonoids. Chin. Pharm..

[48] Tan L.H., Zhang D., Wang G., Yu B., Zhao S.P., Wang J.W., Yao L., Cao W.G. (2016). Comparative analyses of flavonoids compositions and antioxidant activities of Hawk tea from six botanical origins. Ind. Crop. Prod..

[49] Wang J., Lu W.L., Zhang Y.L., Tang M.F., Tang W.J., Li J. (2014). Chemical constituents from *Litsea Coreana* L.. Acta Univ. Med. Anhui.

[50] Yasuda M., Yasutake K., Hino M., Ohwatari H., Ohmagari N., Takedomi K., Tanaka T., Nonaka G. (2014). Inhibitory effects of polyphenols from water chestnut (*Trapa japonica*) husk on glycolytic enzymes and postprandial blood glucose elevation in mice. Food Chem..

[51] Apostolidis E., Lee C.M. (2010). In vitro potential of Ascophyllum nodosum phenolic antioxidant-mediated alpha-glucosidase and alpha-amylase inhibition. J. Food Sci..

